# Mechanism understanding in cryo atomic layer etching of SiO_2_ based upon C_4_F_8_ physisorption

**DOI:** 10.1038/s41598-020-79560-z

**Published:** 2021-01-11

**Authors:** G. Antoun, T. Tillocher, P. Lefaucheux, J. Faguet, K. Maekawa, R. Dussart

**Affiliations:** 1 GREMI, Orléans University-CNRS, 14 Rue d’Issoudun, BP 6744, 45067 Orléans, France; 2 TEL Technology Center, America, LLC, NanoFab 300 South 255 Fuller Rd., Suite 214, Albany, NY USA

**Keywords:** Nanoscience and technology, Physics

## Abstract

Cryogenic Atomic Layer Etching (cryo-ALE) of SiO_2_ based on alternating a C_4_F_8_ molecule physisorption step and an argon plasma step, has been enhanced thanks to a better understanding of the mechanism. First, we used Quadrupole Mass spectrometry (QMS) and spectroscopic ellipsometry analyses to evaluate the residence time of physisorbed C_4_F_8_ molecules versus temperature and pressure on SiO_2_ surface. QMS monitoring of the SiF_4_ etching by-product also enabled to follow the self-limiting etching behavior. Finally, a SiO_2_ cryo-ALE process was proposed at a temperature of − 90 °C resulting in a very linear etch over 150 cycles and an Etch amount Per Cycle as low as 0.13 nm/cycle.

## Introduction

In the area of nanotechnology and sub-10 nm devices, Atomic Layer Etching (ALE) has become one of the most promising processes to overcome the latest and greatest challenges. More specifically, selective etching of silicon dioxide over other materials such as silicon or silicon nitride have attracted interest from many researchers^1–5^. One of the main solutions to perform anisotropic ALE of SiO_2_ is to use fluorocarbon-based plasmas to deposit a very thin FC modified layer on the surface. This layer can then be etched using Ar or O_2_ plasma at low ion energy bombardment^2,3,6–11^. However, some drifts were reported in the processes, with an increase of the etch amount per cycle (EPC) due to the fluorine contamination of the reactor walls^8–10,12^. Yet, *Dallorto *et al. have shown that the effect of fluorine from the reactor wall contamination is reduced by decreasing the substrate temperature to − 10 °C and below^12^. More generally, cryogenic processes demonstrated to be clean processes with limited chamber wall contamination^13^.

Therefore, cryo-Atomic Layer Etching (Cryo-ALE) is proposed as an alternative to etch SiO_2_ in fluorocarbon-based chemistry but without plasma during the deposition step. In this process, the substrate is cooled to a temperature below − 80 °C. A C_4_F_8_ gas is injected and molecules adsorb on the cooled substrate surface. After purging the gas, an Ar plasma is initiated to activate the etching by low energy ion bombardment. A proof of principle has been previously published^14^, showing that cryo-ALE based on C_4_F_8_ physisorption was working at − 120 °C in our experimental conditions. However, etching was suppressed at − 110 °C, owing to the desorption of C_4_F_8_ from the substrate surface being too fast if the temperature is not low enough. Thus, at temperatures below − 110 °C, a self-limiting regime was achieved and an etch per cycle as low as 0.4 nm was obtained. With this process, fluorocarbon polymer is no longer deposited on the reactor walls significantly reducing chamber contamination and limiting process drift. As a result, it is easier to control the etching through many etch cycles.

In this article, we report on the residence time of C_4_F_8_ versus temperature and pressure. Both ellipsometry and mass spectrometry measurements are used to characterize the adsorption and desorption of C_4_F_8_ from the SiO_2_ surface. The improved understanding of the C_4_F_8_ residence time enabled for a significant extension of the SiO_2_ cryo-ALE process temperature range towards higher and more practical temperature.

## Experimental methods

In order to understand the adsorption of C_4_F_8,_ 150 mm SiO_2_ carrier wafers are used, on which SiO_2_ coupons are glued. The SiO_2_ coupons are composed of (100) silicon samples with 100 nm thick thermal silicon dioxide layer. A special glue material is spread uniformly on the backside of the sample and is stable at very low temperature. It has a very good thermal conductivity and is easily removed after use.

As the coupons consist of thin films of SiO_2_ on silicon, they enable a very accurate fit by ellipsometry at the nanoscale.

**Figure 1:**
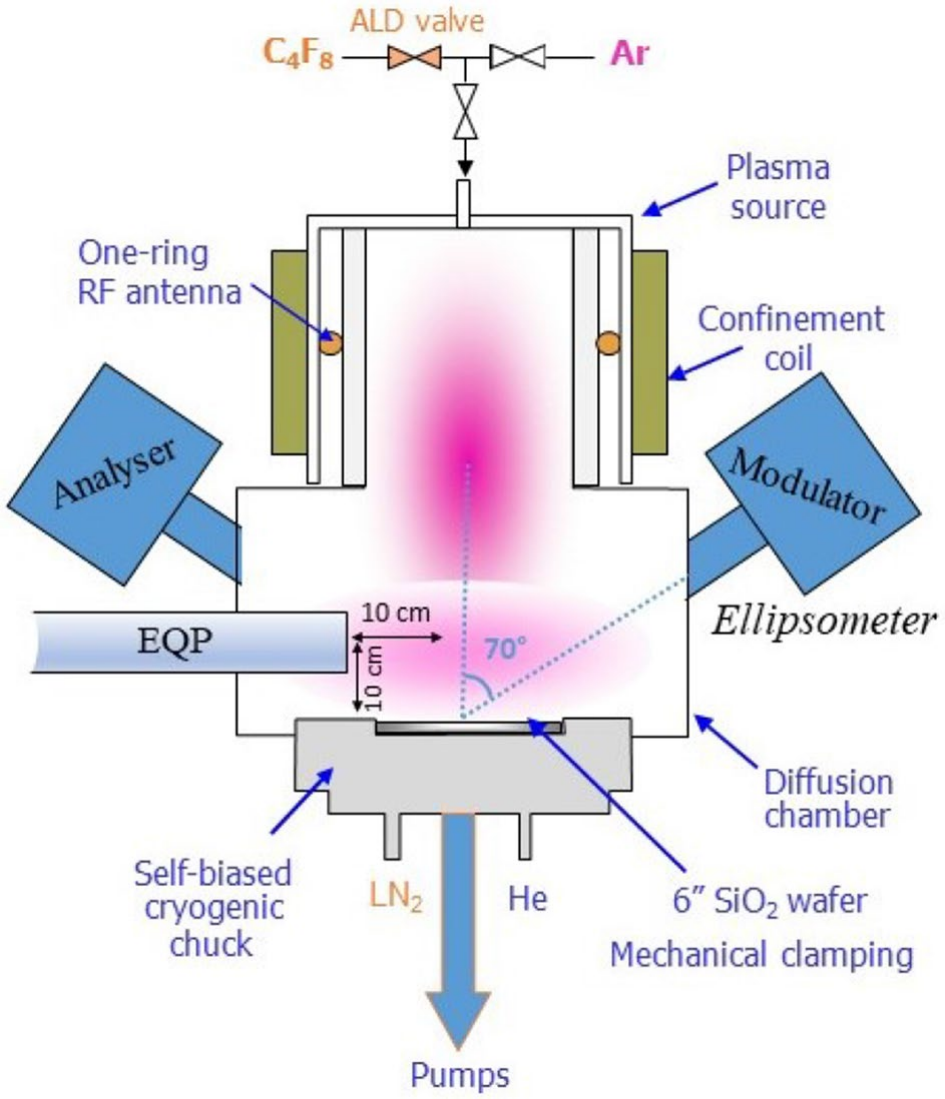
Sketch of the cryogenic ICP reactor equipped with QMS and SE.

The prepared samples are then introduced into an inductively coupled plasma reactor that is equipped with a diffusion chamber and a cryogenic substrate holder. The chuck can be cooled down by liquid nitrogen and temperature is controlled and stabilized using a Proportional Integral Derivative (PID) system. Wafers are mechanically clamped and a backside helium pressure provides for an optimal thermal contact with the chuck. A sketch of the reactor can be seen in Fig. 1.

A Horiba Jobin Yvon UVISEL spectroscopic ellipsometer is coupled to the reactor at an incidence angle of 70° to characterize the substrate surface in-situ. It monitors the thickness variation in kinetic mode at the sample surface during the cycles. The sampling interval is set at 2 s, with an integration time of 0.5 s. For each point of acquisition, spectra are acquired using 32 wavelengths from UV to visible. Lorentz and Drude models were then used to model and fit the ellipsometric spectra.

An Electrostatic Quadrupole Mass Spectrometer (QMS) from Hiden Analytical is used in Residual Gas Analysis (RGA) mode to analyze and monitor the species produced or injected in the reactor during the different steps of the ALE cycles. The mass spectrometer can be used in spectrum mode or in Multiple Ion Detection (MID) mode. The electron energy in the ionization chamber is 70 eV. The QMS entrance slit was positioned at 100 mm above the sample and at 100 mm from the center of the reactor. C_2_F_4_^+^ mass (100 amu) corresponds to one of the main peaks from the fragmentation spectrum of C_4_F_8_^15^. Therefore, its signal was acquired to characterize the kinetics of C_4_F_8_ molecules in the chamber. The SiF_3_^+^ line intensity at 85 amu was also recorded, during an ALE process, in order to follow the evolution of SiF_4_ molecules in the reactor, especially during the etch step. SiF_3_^+^ is reported to be the main fragment ion from SiF_4_^16^. The intensity of the detected ions is expressed in counts per second (c/s).

## Results

### Atomic layer etching process at low substrate temperature

**Figure 2:**
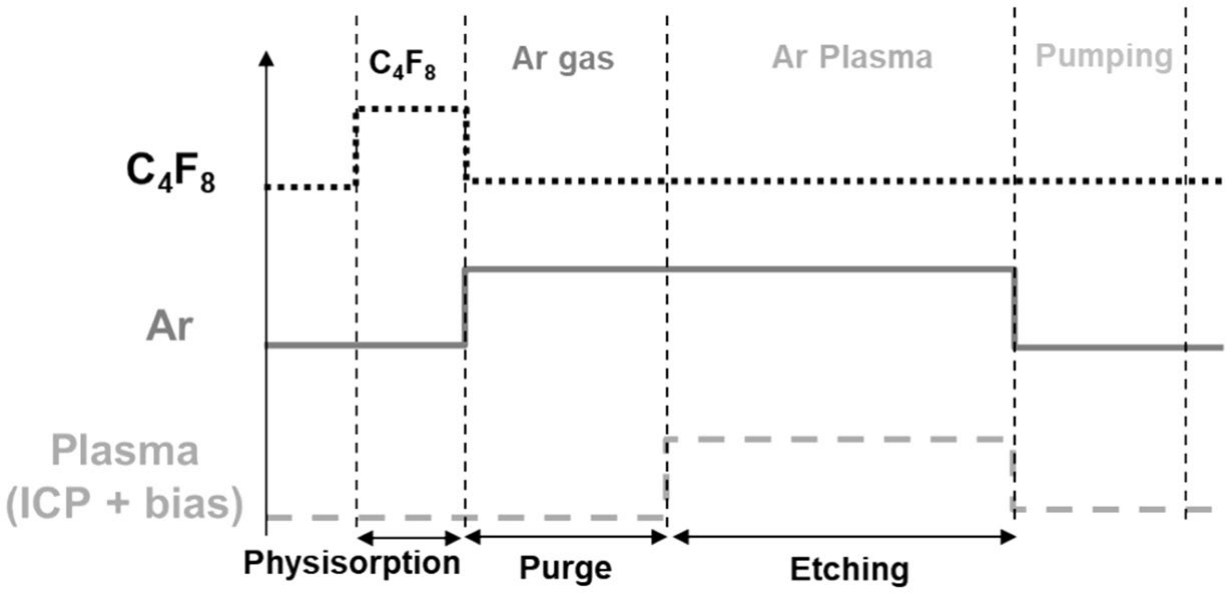
Generic time chart of one cryo-ALE cycle.

In order to characterize and understand the mechanisms involved in Atomic Layer Etching at low substrate temperature, a cryo-ALE process was performed. The generic time chart of cryo-ALE used in^14^ is shown as a reminder in Fig. 2.

**Figure 3:**
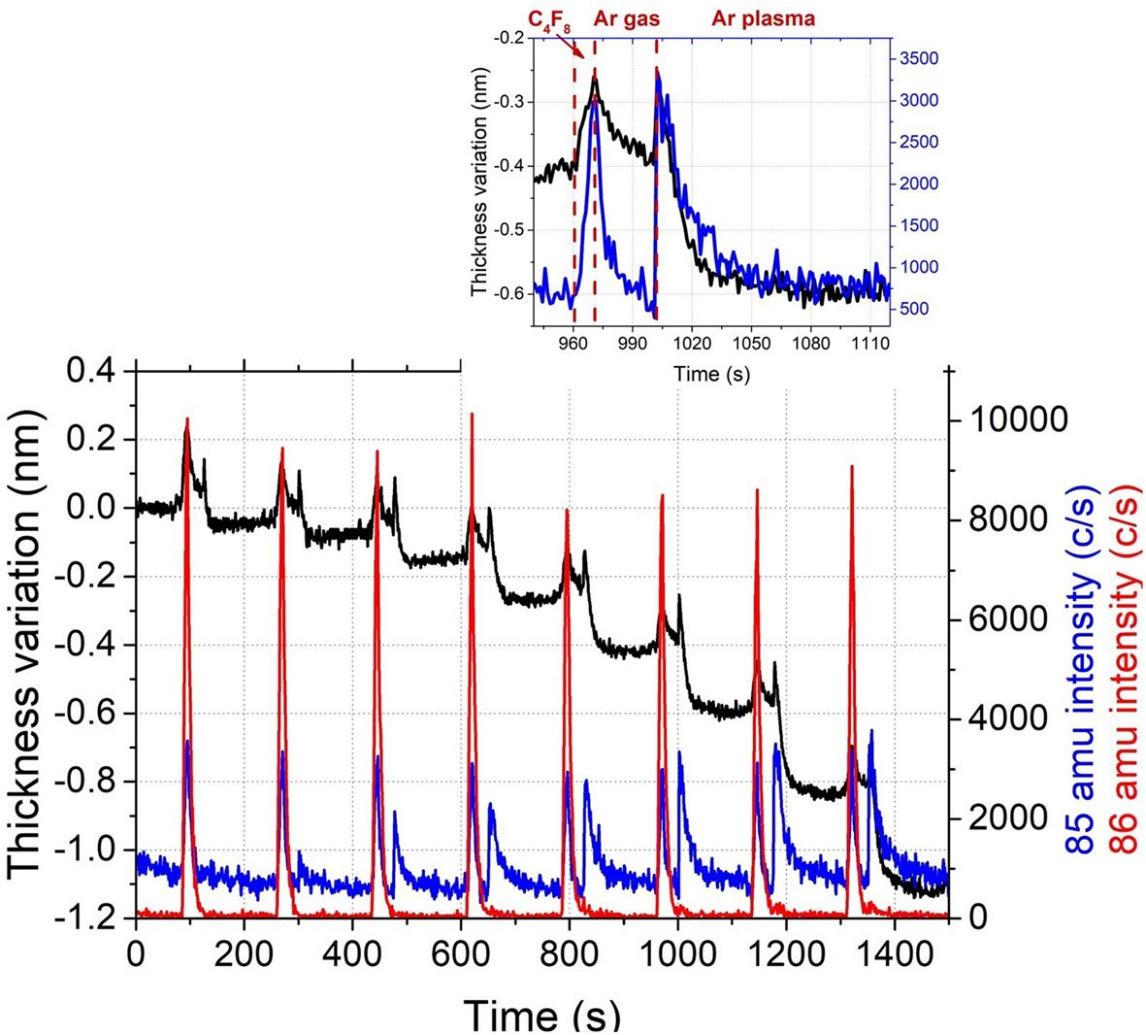
Thickness variation measured by ellipsometry and 85 and 86 amu signals by QMS for 8 ALE cycles performed on SiO_2_, (inset) zoom on the second cycle. (Experimental conditions: T =  − 120 °C, C_4_F_8_ flow: 10 s, 1.9 Pa, Ar purge: 30 s, 3.3 Pa, Ar plasma: 2 min, 3.3 Pa, Psource = 400 W, Vbias =  − 20 V, pumping: 15 s, 0 Pa).

Figure 3 presents 8 cycles of cryo-ALE monitored by in-situ ellipsometry for the thickness variation and by QMS to follow 85 amu corresponding to SiF_3_^+^ signal, which represents SiF_4,_ the main etching by-product. During this process, a C_4_F_8_ gas flow is first injected for 10 s in order to allow for the molecules to physisorb on the cooled substrate surface (− 120 °C). Then, an Ar purge of 30 s is performed in order to remove C_4_F_8_ molecules from the chamber. After that, the Ar plasma is ignited to activate the etch and sustained for 2 min to ensure self-limiting etching is achieved. Finally, a pumping step is performed before starting the next cycle, in order to evacuate all the etching by-products from the chamber. The physisorption step is clearly identified by ellipsometry measurements, as well as by the QMS signal. The SiF_3_^+^ mass of 85 amu is close to the mass of C_4_F_2_^+^ (86 amu) which is an ion from the fragmentation of C_4_F_8_. Hence, as shown in Fig. 3, the first peak of a cycle, observed on QMS signal during the C_4_F_8_ injection is related to C_4_F_2_^+^. When the Ar plasma is initiated, etching is observed by ellipsometry as well as by QMS. During the first three cycles, an increase in SiF_3_^+^ signal and the EPC is observed, before it reaches a steady amount. This shows that the first cycles are in transient state. Surface contamination may limit the etching during these cycles, as well as the formation of a mixing layer such as SiOCF on the surface instead of completion of etching^4,7,17^. Once this layer is formed, the etch amount per cycle becomes constant and self-limiting etching is reached. Then, during the following cycles, the SiF_3_^+^ signal steeply increases the first few seconds, and then starts to decrease (see the inset of Fig. 3). This behavior shows that self-limiting etching is almost reached, inducing less etching and less SiF_4_ by-products. The presence of SiF_3_^+^ signal clearly indicates that the substrate is etched chemically. Indeed, the presence of the self-limiting etching (SLE) is evidenced from the observation of both the film thickness and the SiF_3_^+^ signal simultaneous transition to a plateau, confirming that Ar sputtering does not participate to the etching mechanism. Moreover, the same test, performed at − 110 °C, shows no etching, as reported in^14^, as all C_4_F_8_ molecules desorb from the surface before the ignition of the argon plasma.

### Characterization of the physisorption

#### Temperature dependency

In order to optimize the duration of the argon purge step before plasma initiation, the residence time of the C_4_F_8_ molecules was studied as a function of the setpoint temperature. The C_4_F_8_ gas was injected in the reactor chamber for 1 min at constant pressure (3 Pa) and different temperatures of the SiO_2_ substrate. Then the gas was evacuated during several tens of minutes.

**Figure 4:**
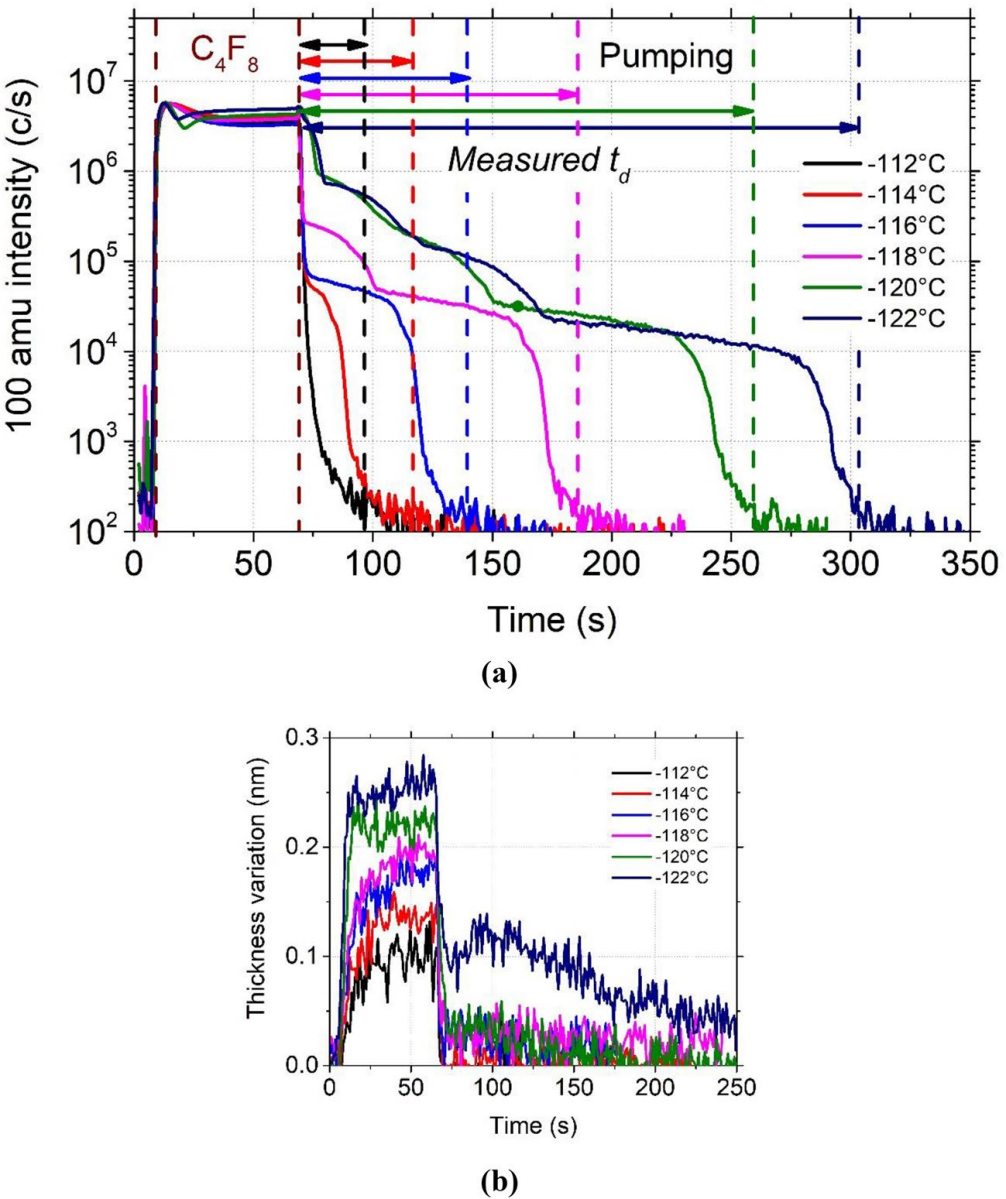
C_4_F_8_ physisorption on SiO_2_ depending on setpoint temperature and monitored (**a**) by QMS by following C_2_F_4_^+^ ion peak intensity evolution and (**b**) by ellipsometry following the thickness variation , both versus time. (Experimental conditions: 1 min C_4_F_8_ flow, 3 Pa followed by a pumping step).

Figure 4a shows the C_2_F_4_^+^ line intensity measured by mass spectrometry for various setpoint temperatures between − 112 and − 122 °C. As soon as C_4_F_8_ gas is injected, the C_2_F_4_^+^ signal increases rapidly and stabilizes for a few seconds after injection as the flow is continuously maintained. When the C_4_F_8_ flow is stopped after 1 min of injection, a sharp drop is first observed for all temperatures. Then, different trends are observed depending on the setpoint temperature. At − 112 °C, the intensity of C_2_F_4_^+^ drops quickly down to a value below 10^3^ c/s. At − 114 °C, the signal drops down to 8 × 10^4^ c/s, decreases slowly and drops again after about 20 s. At − 120 °C, two different kinetics are observed with two shoulders on the curve: after the C_2_F_4_^+^ first drop, a first plateau is reached at 2 × 10^4^ c/s, followed by a second decrease and a second plateau. These different drops and plateaus observed in the C_2_F_4_^+^ line intensity correspond to different desorption steps of C_4_F_8_.

**Figure 5:**
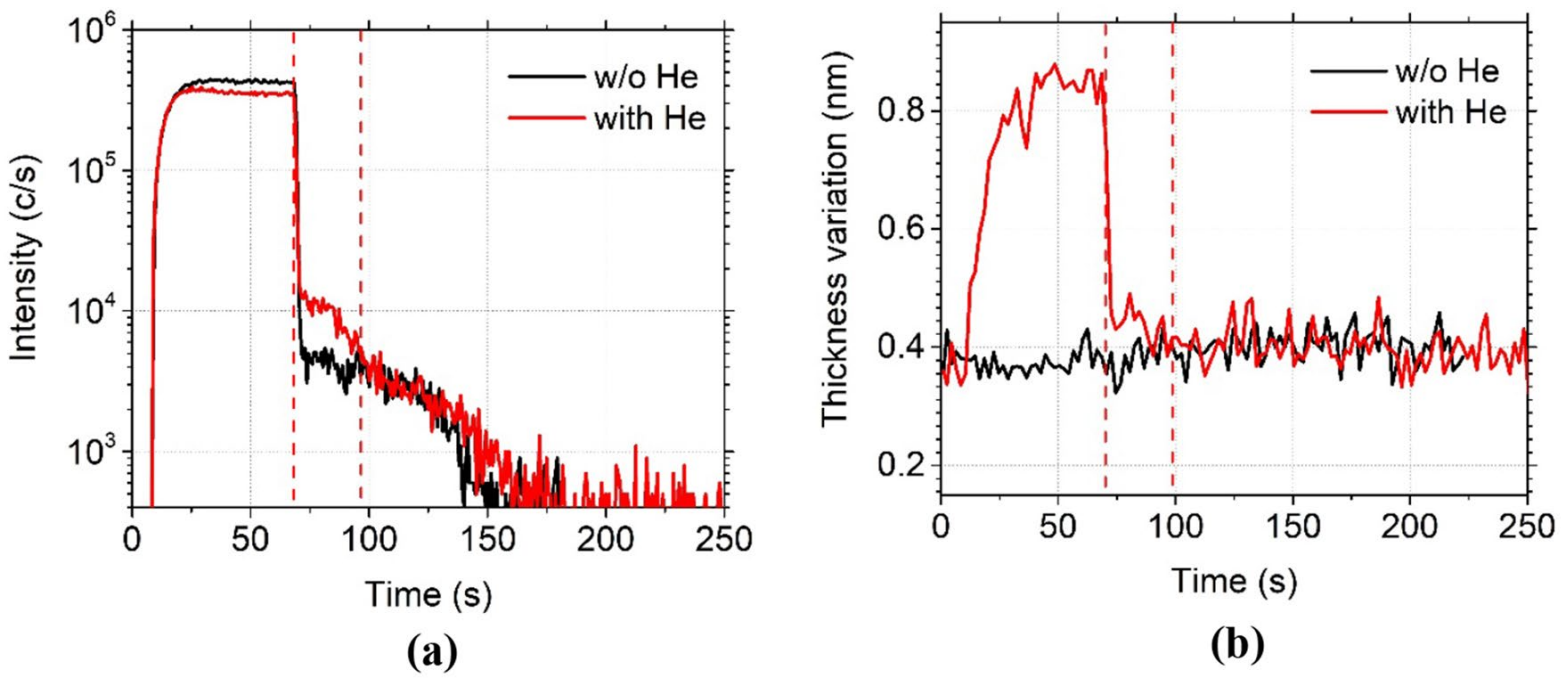
C_4_F_8_ physisorption on SiO_2_ depending on the presence of He backside cooling during the clamping and monitored (**a**) by QMS by following C_2_F_4_^+^ ion peak intensity evolution and (**b**) by ellipsometry following the thickness variation , both versus time. (Experimental conditions: T =  − 120 °C, 1 min C_4_F_8_ flow, 3 Pa followed by a pumping).

To confirm the origin of the first desorption stage, C_4_F_8_ was first physisorbed at − 120 °C and then, its desorption was monitored by QMS and ellipsometry. The test was run with and without helium injection between the chuck and the wafer. Without helium, the wafer is not efficiently cooled and nearly no adsorption should occur on it. The results, illustrated in Fig. 5a. show a clear difference between the two cases: the first desorption stage, between 75 and 100 s (indicated in dashed line in Fig. 5a) does not appear when backside helium flow is not used. In Fig. 5b, ellipsometry data confirms that C_4_F_8_ is not adsorbed without helium. With helium, the thickness grows during the adsorption of C_4_F_8_, then drops when the flow is stopped. However, a small shoulder can be noticed, between 75 and 100 s (indicated in dash line in Fig. 5b), representative of C_4_F_8_ desorption. The difference observed with and without helium shows that the first plateau after the main drop corresponds to the desorption of C_4_F_8_ from the wafer. The other plateaus correspond to desorption of C_4_F_8_ from other parts of the chuck, which are cooled as well but have a slightly lower temperature.

Adsorption kinetics differ from the first adsorption layer to the following ones. Kinetics for the first adsorbed layer and coverage are described by Langmuir theory. Then, each particle in the first layer may be an adsorption center for further adsorbates for the next layers. This multimolecular adsorption kinetic is covered by the BET (Brunauer, Emmett and Teller) theory ^17^.

Several layers can adsorb on the wafer surface, and each layer has its specific residence time. The desorption rate decreases with decreasing temperature as the residence time at the surface increases^18^. In the case of those experiments (Fig. 4), according to spectroscopic ellipsometry measurements, the surface coverage is of the order of one or two monolayers at − 120 °C and higher temperatures. Consequently, Langmuir model can be applied. For lower temperatures, several layers can adsorb to the surface. However, the desorption rate of top layers is usually much higher than the one of the adsorbed layer on SiO_2_.

**Figure 6:**
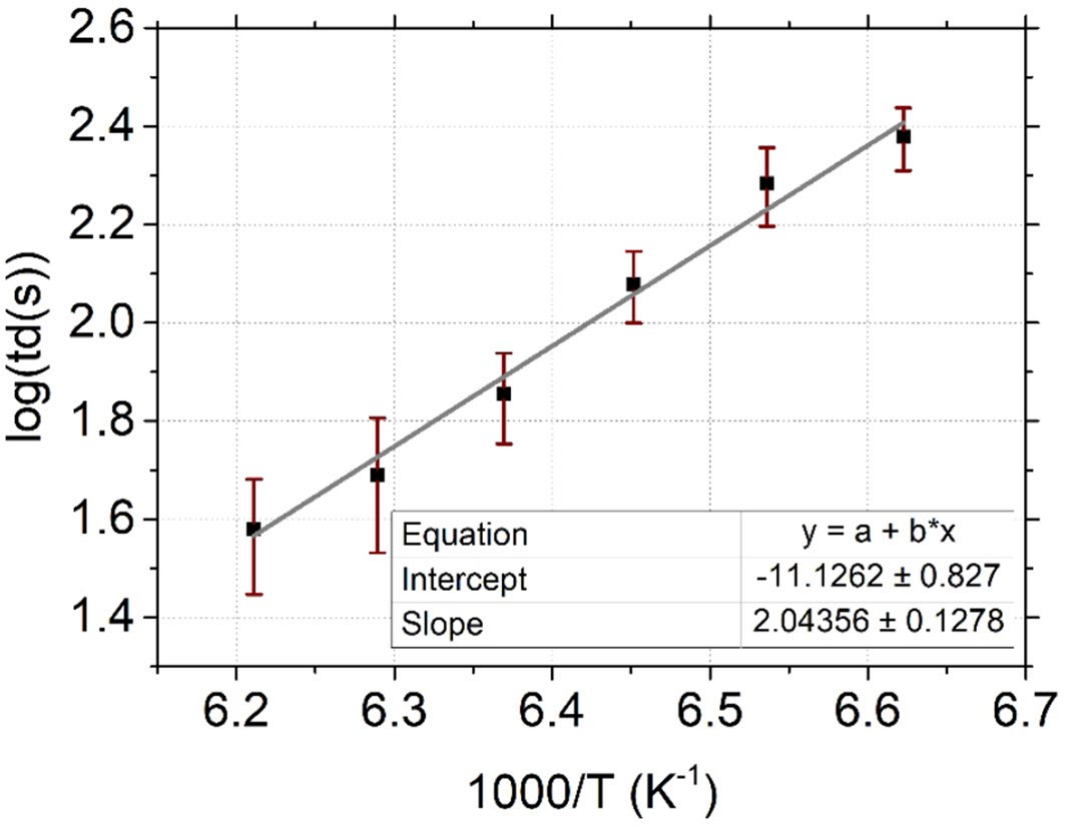
C_4_F_8_ desorption rate depending on temperature, for a C_4_F_8_ injection at 3 Pa.

Consequently, from those hypotheses and based on the results obtained by QMS, presented in Fig. 4a, we can consider that the C_4_F_8_ molecules residence time t_d_ is the delay between the C_4_F_8_ injection stop and the end of the first slope (Fig. 4a). t_d_ was plotted as a function of temperature in Fig. 6. As those measures are extracted from the QMS curves, an error of a few seconds is considered per data point. Then, log (t_d_) was plotted versus 1000/T and fitted using a linear function. It gave a consistent result with the equation of Frenkel-Arrhenius that enables the determination of surface residence time. The equation being:

1 $$t_{d} = t_{d}^{0} exp^{{E_{d} /k_{B} T}}$$

with $t_{d}$ the residence time (s), $t_{d}^{0}$ is the attempt time of the particle for desorption (s), *E*_*d*_ is the energy to enable the desorption (kJ mol^−1^), *k*_*B*_ is the Boltzmann constant (kJ K^−1^) and *T* the substrate temperature (K)^18–20^. From Eq. (1) and the values obtained with the curve fit, the values of ${t}_{d}^{0}$ and *E*_*d*_ were determined to be respectively, 1 × 10^−11^ s and 0.406 eV (39.1 kJ mol^-1^). The desorption energy is very low and is of the order of magnitude of typical binding energy for physisorption.

This graph is of interest to design a cryo-ALE process at different temperatures, especially to define the maximum purge duration between C_4_F_8_ gas injection and the plasma initiation.

#### Pressure dependency

**Figure 7:**
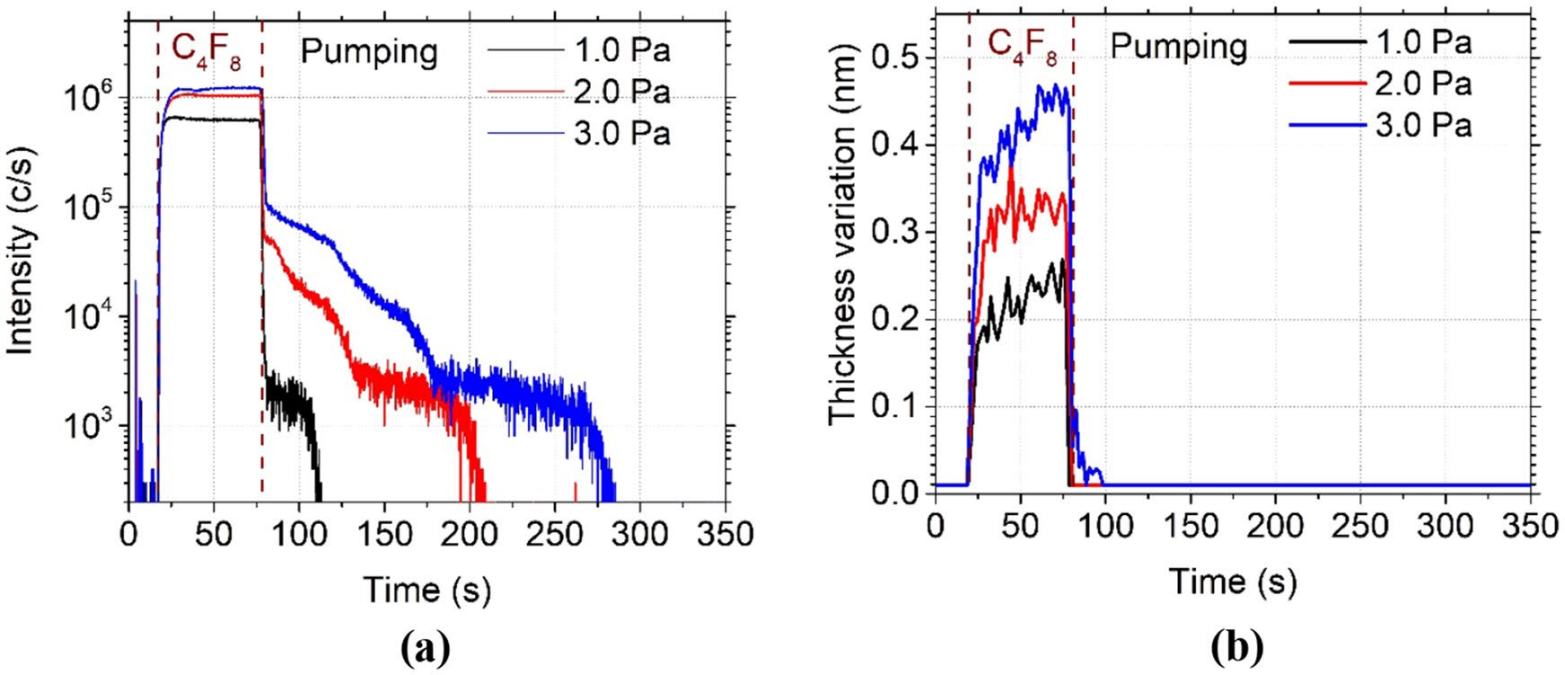
C_4_F_8_ physisorption on SiO_2_ depending on pressure and monitored (**a**) by QMS by following C_2_F_4_^+^ ion peak intensity evolution and (**b**) by ellipsometry following the thickness variation , both versus time. (Experimental conditions: T =  − 120 °C, C_4_F_8_ flow: 1 min followed by a pumping).

The influence of C_4_F_8_ gas pressure was derived from similar tests obtained by injecting C_4_F_8_ gas for 1 min at different pressures at a temperature set to − 120 °C. The results are shown in Fig. 7. Different desorption rates are observed when increasing the pressure. In fact, by increasing the pressure, the density of C_4_F_8_ molecules in the reactor is higher and the quantity of physisorbed molecules is increased. This is also observed by ellipsometry measurements, (Fig. 7b) which show that the thickness of the physisorbed layer increases with pressure. Therefore, it takes a longer time to remove the C_4_F_8_ molecules at a constant desorption rate. Indeed, according to the expression (1) reported in the previous part, the desorption rate does not depend on the gas pressure, but on temperature only.

#### Water influence

**Figure 8:**
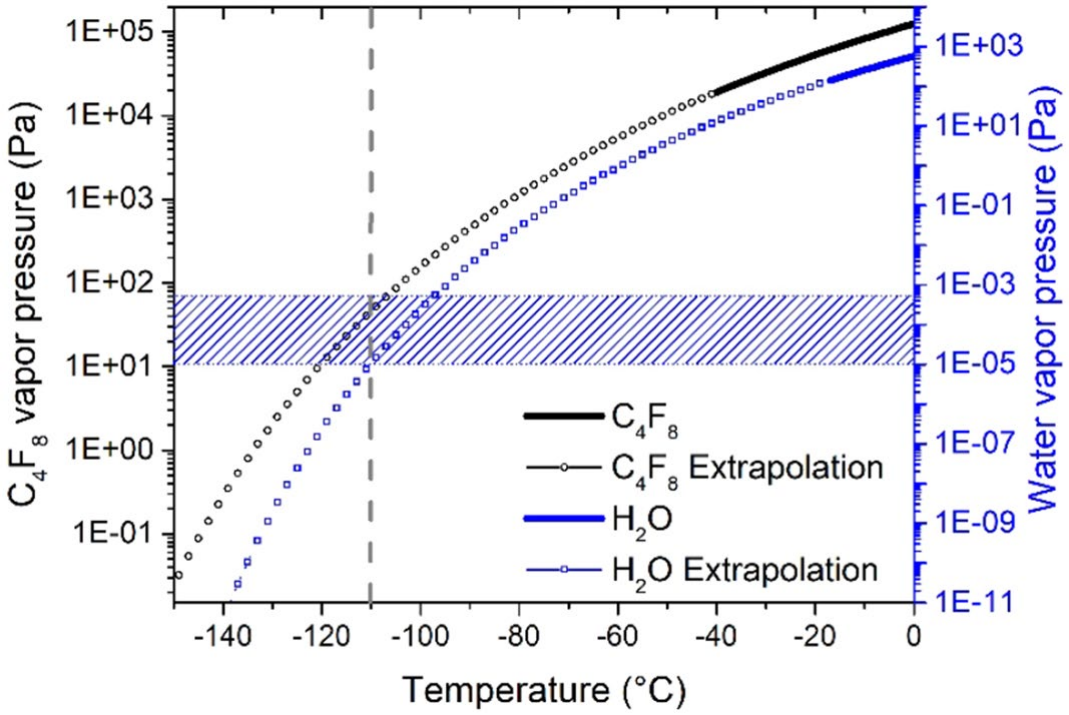
Vapor pressure curves for H_2_O and C_4_F_8_.

For a better understanding of the role of residual water molecules, the vapor pressures curves of C_4_F_8_ and water were plotted together in Fig. 8. In order to cover the temperature range of interest, an extrapolation was performed using Antoine equation parameters from NIST database^21,22^. Hence, in Fig. 8, the part of the curves in bold are from the database and the dotted part lines are extrapolated. The air leakage of the reactor chamber was evaluated at around 0.1 sccm from which it was possible to estimate the water partial pressure during the C_4_F_8_ gas injection. At 3 Pa, the water partial pressure is about 4.3 × 10^−4^ Pa, decreasing to 1.0 × 10^−5^ Pa during the pumping. The striped section in Fig. 8 represents the range of water partial pressure in the process conditions. At temperatures higher than − 110 °C, the effect of water is not significant for C_4_F_8_ physisorption, as in these conditions, water does not condensate (on the right of the water curve).

However, for lower temperatures, the parameters are such that water condenses (on the left side of the water curve).

**Figure 9:**
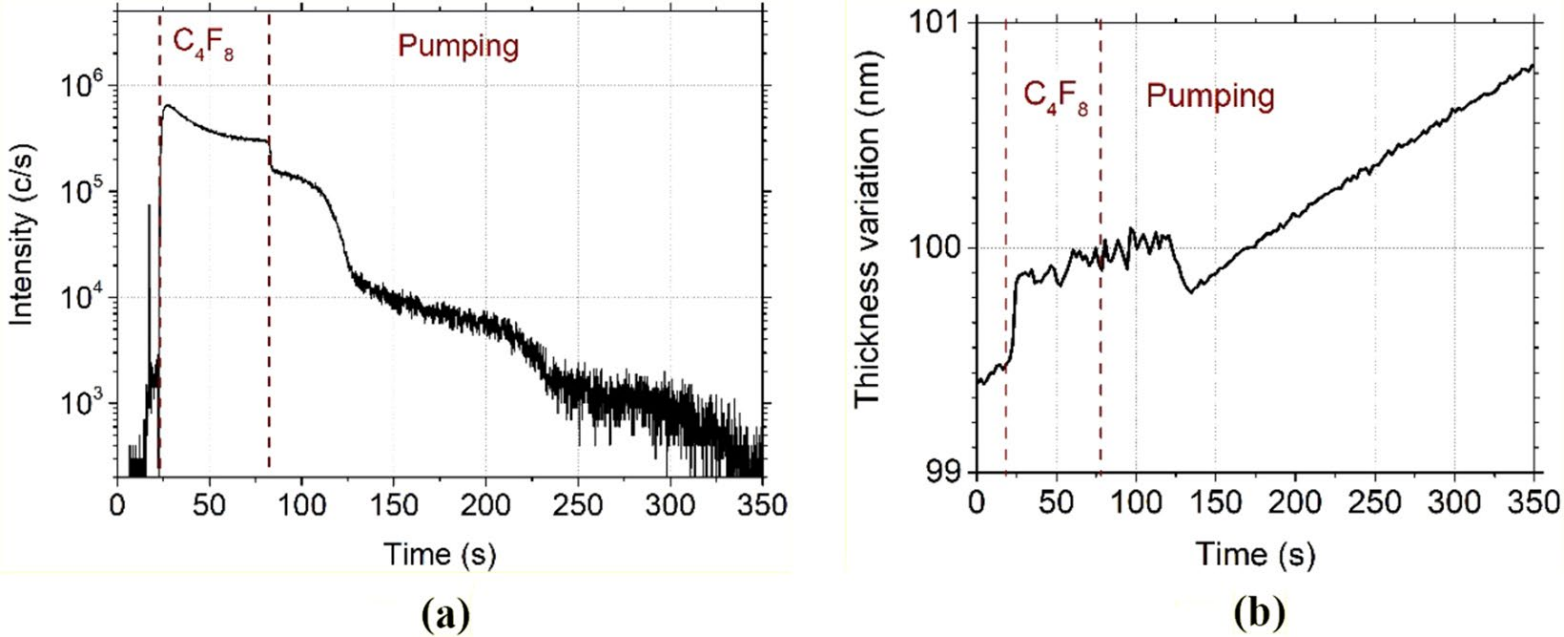
C_4_F_8_ physisorption on SiO_2_ at − 130 °C monitored (**a**) by QMS by following C_2_F_4_^+^ ion peak intensity evolution and (**b**) by ellipsometry following the thickness variation , both versus time. (Experimental conditions: T =  − 130 °C, 1 min C_4_F_8_ flow, 3 Pa followed by a pumping step).

This behavior is observed in Fig. 9 below where physisorption test was performed at − 130 °C. During the C_4_F_8_ injection in Fig. 9a the C_2_F_4_^+^ signal does not stabilize, but rather decreases although the C_4_F_8_ flow is maintained constant inside the chamber. At this temperature, the cooled substrate acts like a cryogenic pump, and the amount of C_4_F_8_ molecules in the chamber is reduced as they start to condense on the cooled surface of the substrate. This is confirmed by ellipsometry measurements in Fig. 9b: the adsorbed film thickness increases during the C_4_F_8_ injection step instead of reaching a plateau as at higher temperatures (Fig. 4b). This result is consistent with the Antoine’s curve giving the vapor pressure of C_4_F_8_ as a function of temperature. By decreasing the temperature, conditions are closer to condensation. Moreover, the point located at T =  − 130 °C and P = 3 Pa is clearly in the condensation part of the water curve. At − 130 °C, water molecules, that are present in the chamber, as evidenced by the continuous signal increase after C_4_F_8_ is pumped out, start to condense on the substrate surface. Consequently, it prevents from observing the signal saturation as expected from the C_4_F_8_ physisorption tests in Fig. 4.

### Optimization of the process

In the letter dedicated to Cryo-ALE^14^, we demonstrated the importance of working at low enough temperature (close to − 120 °C) to reach an etching regime. However, this temperature operating range is often unwanted because it requires the use of liquid nitrogen.

QMS results presented above gave us a better understanding of the mechanisms, enabling the enhancement of the process. The purpose of the following part is to increase the process working temperature.

#### Influence of purge step

**Figure 10:**
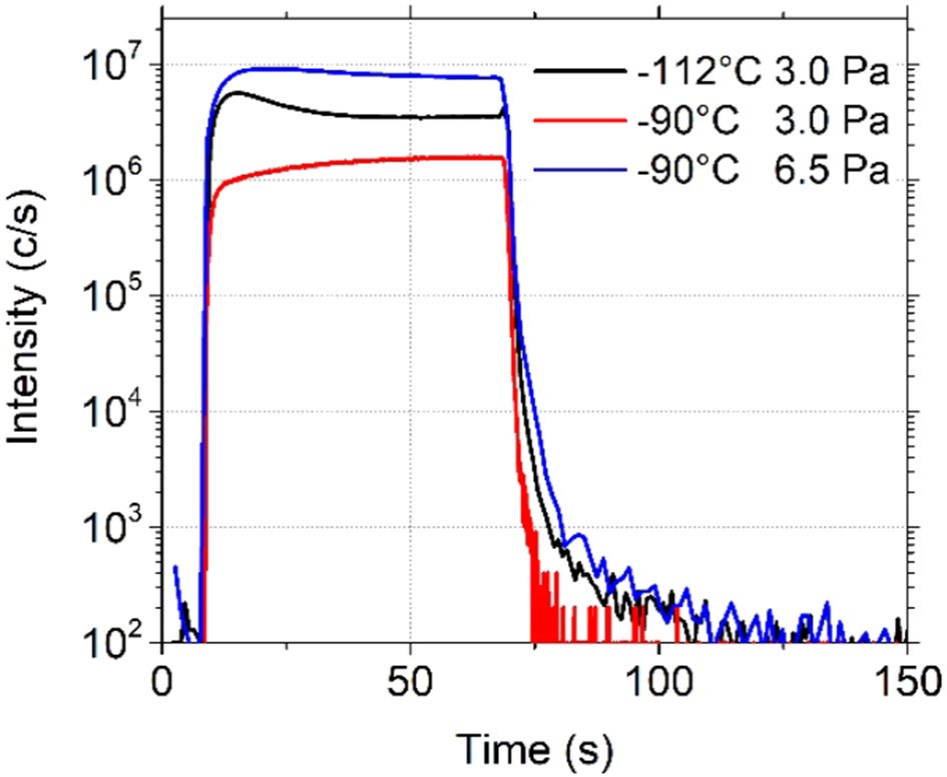
C_4_F_8_ physisorption on SiO_2_ depending on pressure and temperature and monitored by QMS by following C_2_F_4_^+^ ion peak intensity evolution versus time. (Experimental conditions: T =  − 90 °C and − 112 °C, C_4_F_8_ flow: 1 min followed by a pumping).

According to the physisorption tests results in Fig. 7, when the pressure during the injection of C_4_F_8_ is increased, the amount of C_4_F_8_ molecules that physisorb also increases. Again, this is confirmed in Fig. 10 below, where physisorption tests have been repeated at − 90 °C at two different pressures and C_2_F_4_^+^ peak was followed by QMS. It shows that at 3.0 Pa, C_2_F_4_^+^ signal decreases sharply when C_4_F_8_ is stopped. Whereas, few additional seconds are needed at 6.5 Pa to remove all C_4_F_8_. By comparing this latter with the test at − 112 °C at 3.0 Pa, it is possible to observe that the residence time of C_4_F_8_ at − 90 °C 6.5 Pa is almost equal to the residence time at − 112 °C at 3.0 Pa. This confirms that increasing the pressure will help processing at higher temperatures.

**Figure 11:**
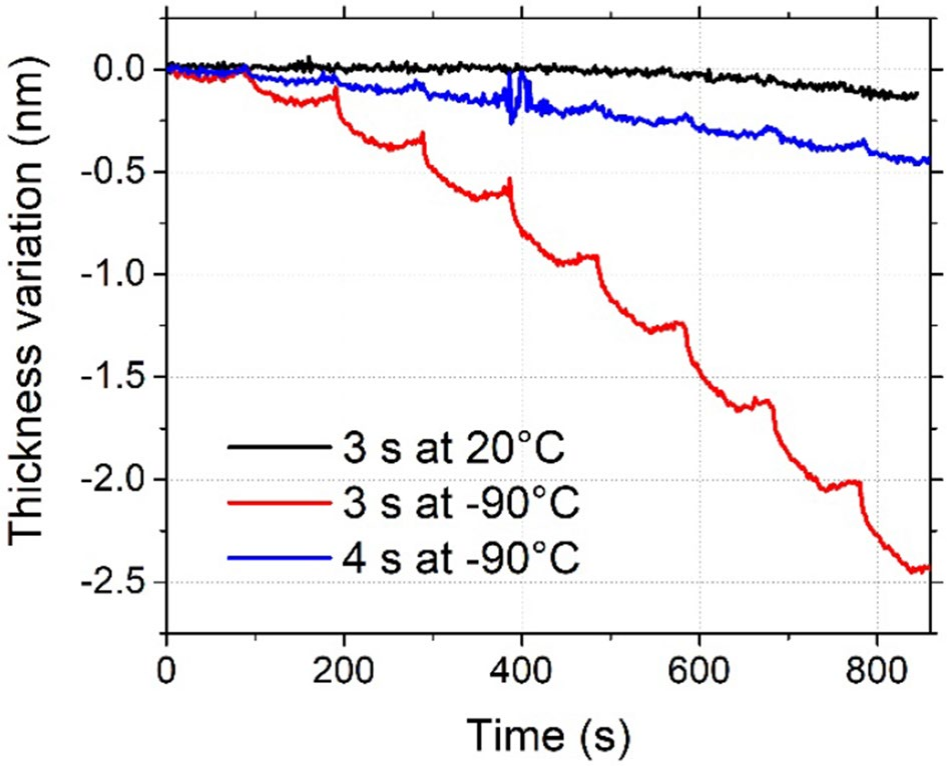
8 ALE cycles performed on SiO_2_ with the thickness variation followed by ellipsometry. (Experimental conditions: T = 20 °C/ − 90 °C, C_4_F_8_ flow: 20 s, 6 Pa, Ar purge: 3 s/3 s/4 s, < 1 Pa, Ar plasma: 1 min, 3 Pa, Psource = 400 W, Vbias =  − 20 V, pumping: 15 s, < 1.0 × 10^−3^ Pa).

In Figs. 8,  11 cryo-ALE cycles were performed at − 90 °C. The pressure of C_4_F_8_ needed to be raised to 6 Pa, which is higher than the usual pressure processing that was used in previous experiments (~ 2–3 Pa), to enable the physisorption of few monolayers of C_4_F_8_ molecules. However, the subsequent purge step time is also very critical. If the purge step time is too long, all C_4_F_8_ molecules desorb before starting the argon plasma for the etching step. If it is too short, C_4_F_8_ gas is not totally evacuated from the reactor chamber and CF_x_ are created in the chamber during the Ar plasma. The process may thus no longer be controlled. As observed in Fig. 11, a clear difference is obtained by varying the purge step time by only 1 s. Very low etching is observed at 4 s purge time because most of C_4_F_8_ molecules have desorbed from the surface. Whereas, if the time is decreased to 3 s, a sufficient quantity of molecules is still present at the surface to allow for the etching of 0.3 nm of SiO_2_ per cycle. The Ar plasma in this process lasts only 1 min, which is on the limits for reaching the self-limiting etching plateau. During this etching plasma, half of the etched amount is removed during the first 15 s.

Same test with 3 s for the purge step was performed at 20 °C. No etching occurs in this case. This proves that the etching achieved at − 90 °C in the same conditions is due to physisorbed species and not from residual C_4_F_8_ in the chamber. It also shows that no sputtering occurs during the Ar plasma.

#### Etch cycles repeatability

**Figure 12:**
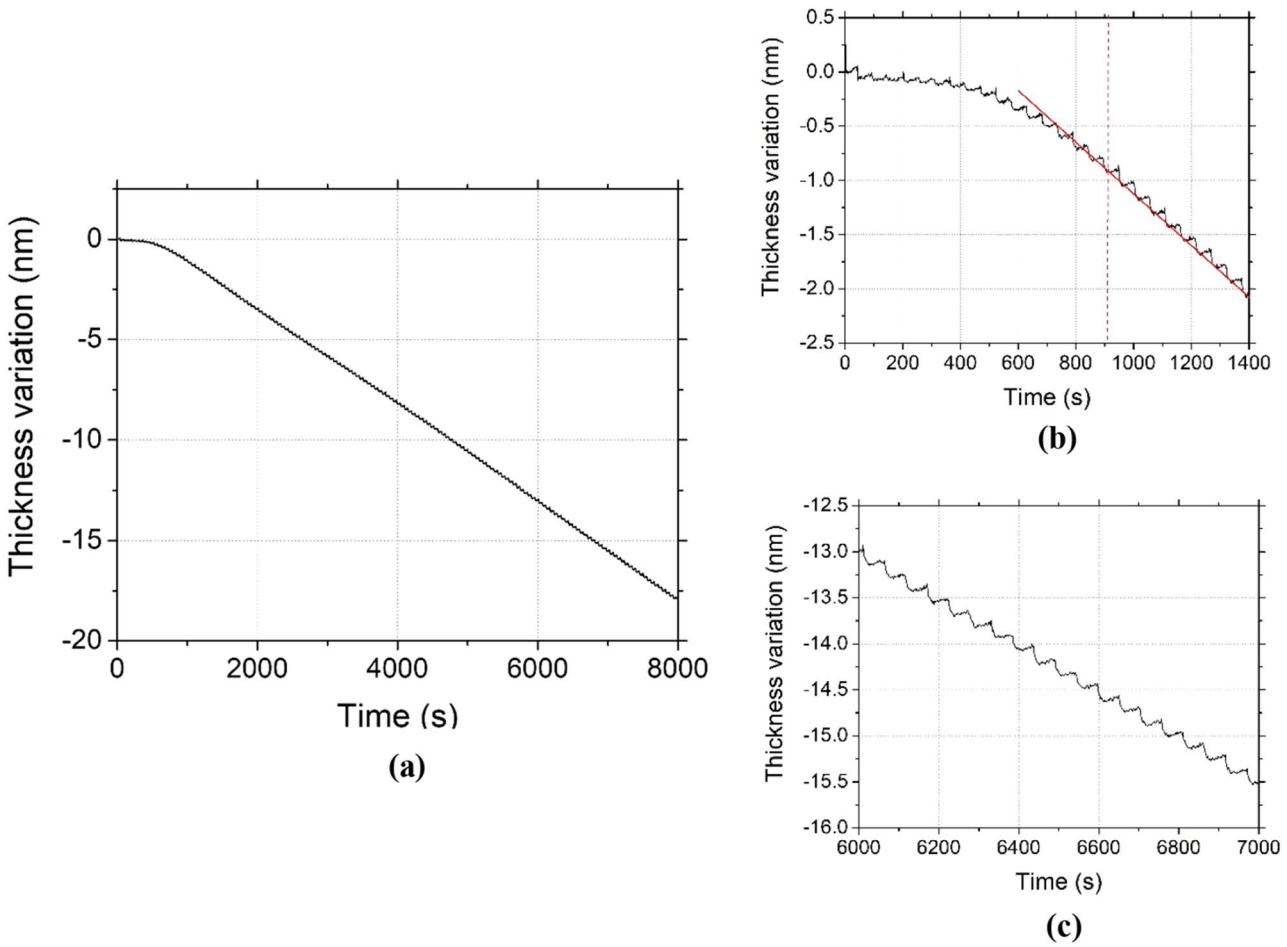
(**a**) 150 ALE cycles performed on SiO_2_, (**b**) zoom on the beginning of the process, (**c**) zoom close to the end of the process. (Experimental conditions: T =  − 90 °C, C_4_F_8_ flow: 20 s, 5.5 Pa, Ar purge: 3 s, < 1 Pa, Ar plasma: 15 s, 3 Pa, Psource = 400 W, Vbias =  − 20 V, pumping: 15 s, < 1.0 × 10^−3^ Pa).

It is essential in the nanotechnology industry to be able to run processes without drifts. To check the repeatability and robustness of the process presented in Fig. 11, 150 cryo-ALE cycles have been performed on SiO_2_ at − 90 °C and monitored by ellipsometry as shown in Fig. 12a. Relying on observations from Fig. 11, the Ar plasma step time for this process has been reduced to 15 s instead of 1 min, in order to reduce the process time. Figure 12b is an inset showing that it takes about 16 cycles before the process reaches a constant etching amount per cycle. Indeed, during those first cycles, the EPC is first close to 0.05 nm/cycle and increases until it becomes stable at 0.13 nm/cycle, until the end of the process (Fig. 12c). The steady etch amount per cycle is in fact reached after having etched 0.5 nm, which corresponds to approximately one monolayer of SiO_2_. During those first cycles, the surface is being modified to form a SiOCF like layer^4,17^. As the temperature is higher than in the previous processes, − 90 °C instead of − 120 °C, less C_4_F_8_ is adsorbing per cycle, and consequently, more cycles are needed before reaching a quasi-steady surface state. Once the first monolayer, which is also expected to be contaminated by carbon^7^, is removed, the surface modification remains the same at the beginning of all the cycles: the SiO_2_ layer should contain the same amount of active sites for C_4_F_8_ physisorption. Moreover, the absence of drift supports that processes based on physisorption at low substrate temperature limit reactor wall contamination and hence the occurrence of drifts. The surface roughness remains the same before and after etching and is close to 0.40 nm for the Root Mean Square roughness (Rq).

## Conclusion

The proposed cryogenic Atomic Layer Etching of SiO_2_ is a process based on a first injection of C_4_F_8_ gas without plasma, followed by argon plasma to activate chemical reactions between physisorbed molecules and SiO_2_ at the surface. QMS and spectroscopic ellipsometry were used to better understand the parameters affecting physisorption and desorption. The increase of residence time of C_4_F_8_ molecules at the surface when decreasing the setpoint temperature was clearly observed by mass spectrometry. At higher pressure, desorption takes more time due the higher amount of physisorbed molecules. Consequently, the thickness of adsorbed molecules does not reach a plateau as observed at higher temperatures (between − 90 and − 120 °C). From these observations, the operating process temperature has been increased from − 120 to − 90 °C. To this end, the pressure has also been raised from 3 to 6 Pa and the purge step time was decreased to prevent the desorption of all the physisorbed molecules. QMS tests also enabled to monitor the etching and predict reaching of the self-limiting etching by following the SiF_3_^+^ signal.

Finally, a remarkably linear etching has been observed when performing 150 cryo-ALE cycles at − 90 °C. The absence of a process drift shows, that contamination of the reactor wall remains very low in these cryogenic process conditions.
