# Author Correction: Mechanism understanding in cryo atomic layer etching of SiO_2_ based upon C_4_F_8_ physisorption

**DOI:** 10.1038/s41598-022-06291-8

**Published:** 2022-02-01

**Authors:** G. Antoun, T. Tillocher, P. Lefaucheux, J. Faguet, K. Maekawa, R. Dussart

**Affiliations:** 1 GREMI, Orléans University-CNRS, 14 Rue d’Issoudun, BP 6744, 45067 Orléans, France; 2 TEL Technology Center, America, LLC, NanoFab 300 South 255 Fuller Rd., Suite 214, Albany, NY USA

Correction to: *Scientific Reports* 10.1038/s41598-020-79560-z, published online 11 January 2021

The original version of this Article contained an error in the Results section, under the subheading ‘Temperature dependency’ where

“From Eq. (1) and the values obtained with the curve fit, the values of ${t}_{d}^{0}$ and *E*_d_ were determined to be respectively, 1 × 10^−11^ s and 17 kJ mol^−1^ (0.18 eV).”

now reads:

“From Eq. (1) and the values obtained with the curve fit, the values of ${t}_{d}^{0}$ and *E*_d_ were determined to be respectively, 1 × 10^−11^ s and 0.406 eV (39.1 kJ mol^−1^).”

The original Article has been corrected.

